# Simulation-Assisted Augmentation of Missing Wedge and Region-of-Interest Computed Tomography Data

**DOI:** 10.3390/jimaging10010011

**Published:** 2023-12-29

**Authors:** Vladimir O. Alekseychuk, Andreas Kupsch, David Plotzki, Carsten Bellon, Giovanni Bruno

**Affiliations:** 1 Institute of Computer Vision & Remote Sensing, Technical University Berlin, Marchstr. 23, 10587 Berlin, Germany; vladimir.alekseychuk@vision-in-x.com; 2 Vision in X Industrial Imaging GmbH, Johann-Hittorf-Str. 8, 12489 Berlin, Germany; 3 Bundesanstalt für Materialforschung und -Prüfung (BAM), Unter den Eichen 87, 12205 Berlin, Germany; david.plotzki@uni-leipzig.de (D.P.); giovanni.bruno@bam.de (G.B.); 4 Felix Bloch Institute for Solid State Physics, University Leipzig, 04103 Leipzig, Germany; 5 Institute of Physics and Astronomy, University Potsdam, Karl-Liebknecht-Str. 24–25, 14476 Potsdam, Germany

**Keywords:** computed tomography, missing wedge, region of interest, augmented data, CT simulation, aRTist

## Abstract

This study reports a strategy to use sophisticated, realistic X-ray Computed Tomography (CT) simulations to reduce Missing Wedge (MW) and Region-of-Interest (RoI) artifacts in FBP (Filtered Back-Projection) reconstructions. A 3D model of the object is used to simulate the projections that include the missing information inside the MW and outside the RoI. Such information augments the experimental projections, thereby drastically improving the reconstruction results. An X-ray CT dataset of a selected object is modified to mimic various degrees of RoI and MW problems. The results are evaluated in comparison to a standard FBP reconstruction of the complete dataset. In all cases, the reconstruction quality is significantly improved. Small inclusions present in the scanned object are better localized and quantified. The proposed method has the potential to improve the results of any CT reconstruction algorithm.

## 1. Introduction

It is not always possible to perform an optimized X-ray computed tomography (XCT) scan with complete projection angles that cover a whole object. Even if the radiation exposure is perfectly configured, geometrical problems such as a too large object or blocked rotation path may arise. If the used detector is smaller than the object projection, the object is scanned in a limited range, called Region-of-Interest (RoI). While this procedure can be exploited to better visualize features in large samples, it produces strong artifacts at the RoI edge. In some cases, it is not possible to acquire projections from certain angles, the so-called Missing Wedge (MW) problem, which leads to distorted reconstructed objects showing the typical lemon-shaped artifacts.

To mitigate the influence of these artifacts, in this work, we deploy a strategy using a simulated XCT scan resembling the original data and including the missing information in the limited experimental data.

For that approach to work, it is essential to have detailed a priori information in the form of a 3D model of the scanned part. Such information can be produced using an automated measurement system or is already available from the design of a component. The proposed method could improve FBP reconstruction of large parts, such as cast aluminium engine blocks, that cannot be scanned as a whole (i.e., can only be scanned in RoI mode) and may not be accessible from all angles by the XCT system (MW problem).

The solution to the above-mentioned problems has been attempted by several authors. Hsieh et al. [1] augmented missing RoI portions with parts of water cylinders, mimicking the constant attenuation sum of an ideal XCT set-up. Using water as an extrapolation medium works best in a medical context but could be extended to other materials in an NDT (non-destructive testing) context. The RoI must be large enough that at least some projections cover the whole patient to obtain the attenuation sum. Xia et al. [2] focused on creating a 3D model based on two low-dose radiographic projections, usually made to center a patient inside a medical XCT system. A rough 3D volume of the patient was generated by computing boundaries and fitting ellipses slice-wise onto them. The resulting patient model was forward-projected to identify boundaries in the sinogram. When applying known RoI artifact reduction algorithms, such as the water cylinder extrapolation [1], Xia et al. modified them to meet the restriction that the extrapolated data shall not exceed the simulated patient boundaries, resulting in an “error reduction by a factor of 8” [1].

Several approaches to mitigate typical artifacts caused by missing/incomplete data [3,4] have been made through the application of the DIRECTT (Direct Iterative Reconstruction of Computed Tomography Trajectories) [3], developed at BAM, Berlin, Germany. The basic principle is the computation of line integrals along sinusoidal traces, which correspond to each individual pixel position of the tomogram. At each iteration cycle, the predominant pixels are selected, weighted, and added to the intermediate reconstruction of the preceding cycle. Successful curation of artifacts has been achieved at the expense of numerous iterations. Recently, an Artificial Intelligence (AI) approach has been implemented to successfully overcome MW artifacts of cryogenic electron tomography data of viruses [5].

The above-mentioned approaches assume that the characteristics of the inspected object in terms of geometry and/or material are unknown. They rely on mathematical constraints while disregarding the object’s actual characteristics. The higher the percentage of missing data is, the larger the uncertainty of the reconstruction and the lower the image quality becomes.

With some objects, it is possible to rely on detailed a priori knowledge. If an object is manufactured multiple times and a full scan of a reference object is available, one could compare individual projections between the reference and the new scan, even if the new scan lacks some data. Moreover, it would be possible to use the reference image information to fill in the missing portions of the new scan so that a reconstruction is mostly unaffected by missing data artifacts.

If such a full reference scan is unavailable, though, it can be created by using a detailed XCT simulation that considers physical effects such as beam hardening and scattering. Of course, a 3D model of the scanned object needs to be available. The use of simulated reference information to fill missing data portions offers a high degree of flexibility (to fit the experimental scan).

Heußer et al. [6] proposed a simulation-based approach, which employed previous artifact-free medical or database scans, the results of which were non-uniformly distorted to obtain artifact-free projection data near metal implants. Maier et al. [7] used CAD models to create artifact-containing projections of an object to isolate the artifacts from the simulated reconstruction. These isolated artifacts were then used as a correction term for analytical reconstructions of Missing Wedge and Region-of-Interest scenarios.

The novelty of our approach is the use of sophisticated X-ray simulation software, contrary to [6,7,8,9]. We use an ideal 3D model of the scanned object that has usually not been scanned before and may be unique. Focusing on a priori 3D data eliminates the influence of artifacts from a previous reconstruction. Regarding the approach by Maier et al., our method modifies the projection data instead of the reconstruction and focuses mainly on the preservation of small interior material features instead of the general restoration of geometry.

## 2. Materials and Methods

**Figure 1:**
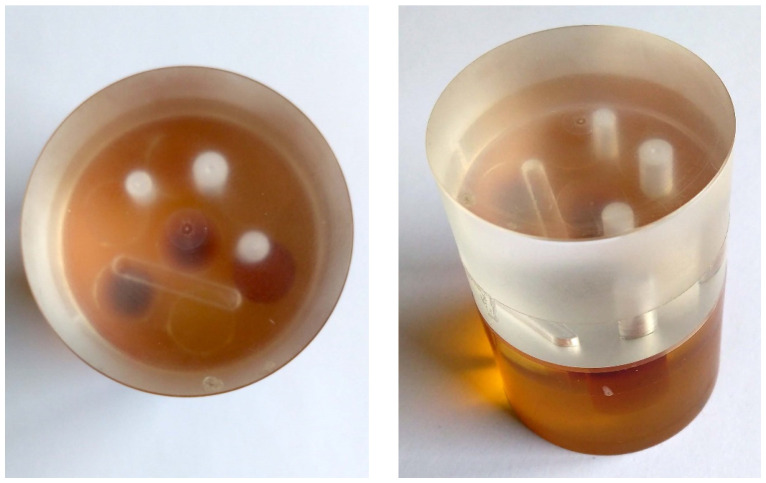
Photographs of the acrylic cylinder specimen show three cylindrical holes of different diameters and a milled channel in the upper (bright transparent) part.

The object under inspection in this study was an acrylic cylinder of approximately 40 mm in diameter and 50 mm in height. It consisted of two parts attached to each other. The center slice in the XCT projections lay just above the connection between the two parts. The upper part contained three cylindrical holes of different diameters and a milled channel with round endings. The lower part had seven cylinders of different materials pressed in it, which will not be considered further here. The cylinder is shown in Figure 1.

The XCT dataset featured 1500 projections that covered 360 degrees of the scan orbit, resulting in angular steps of 0.24 degrees. Each projection had 997 × 1024 pixels in size and was cropped at the sides to center the rotation axis. Additionally, bright (flat field) and dark (beam off) images were acquired for data normalization and detector artifact correction. Further technical data on the used XCT system is listed in Table 1.

For creating simulated CT scan data, the software package aRTist, developed by BAM, Berlin, Germany, was used [10]. It can simulate realistic X-ray projections that consider physical effects such as absorption and scattering, polychromatic spectra of different target materials inside the X-ray source, beam hardening, and the specifics of different types of detectors.

The proposed simulation augmentation was implemented in a custom C++ program, and the reconstruction of the data was realized using the CONRAD framework [11]. It is a plugin for the image processing and analysis tool ImageJ [12]. The reconstruction uses the default FBP pipeline.

Finally, the results were evaluated visually and numerically using the Root Mean Square Error [13], Pearson Correlation Coefficient [14,15], and Structural Similarity Index [16].

## 3. Data Treatment Procedures

### 3.1. Creating Simulation Data

To create any simulated XCT scan, appropriate tools are needed. In the aRTist software [10], the distances of the source, detector, and object to each other need to be specified beforehand. Together with the real scan CT dataset, several files with metadata, such as the geometrical parameters and bright and dark images, were supplied.

Each object inside the simulation also needs to be defined:

- The X-ray source needs to be defined by voltage, current, target and filter material, etc.
- The material of the inspected object needs to be specified. Custom materials can be added such as inputting density and chemical composition.
- The detector needs to be defined by its scintillator material and thickness, target grey value, pixel count and size, etc.

**Figure 2:**
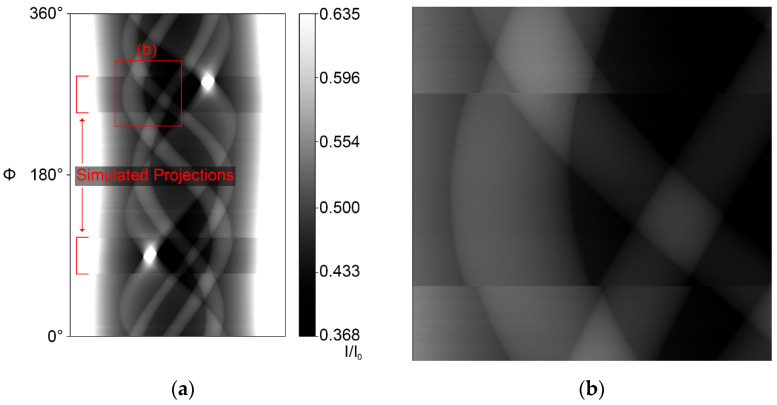
(**a**) Simulation-augmented 40° MW normalized intensity sinogram with imperfect cylinder positioning. (**b**) Enlarged mismatch area. The grey values correspond to the sample transmission.

Next, the settings for scatter simulation need to be set, and a flatfield correction of the image needs to be performed. If the grey values of the simulated image do not correspond to the experimental projections, the characteristic curve of the detector can be manually adjusted. The characteristic curve represents the (non-linear) mapping of absorbed energy intensities onto grey values (see Figure S1 in supplementary material) regarding the individual behavior of each detector pixel. This representation is done through a curve that can be defined by setting at least two points and linear interpolation of the values between them. It was necessary to add several points and interpolate between them because the first defined linear function strongly differed from the target grey values, and the internal parameters of the detector were mostly unknown. An estimation of these parameters resulted in grey value differences of several hundred units, which led to very distinct streaks in the reconstructed image (see Figure 2b). After applying a custom detector characteristic curve, the grey value difference between real and simulated reconstructions could be reduced to a mean of 257 (~13% of mean reconstruction intensity) grey values. The effect of a grey value mismatch that preserves geometry but differs in the values themself is that ROI and MW artifacts are not completely removed but dampened. In the ROI case, a slight ring artifact stays behind, and in the MW case, the corrected edges of internal structures show slight grey value errors in the direction perpendicular to the mean MW direction.

Another important factor for an accurate simulation representation is the correct positioning of the virtual object in the 3D simulation space. The 3D model itself was created with the CAD software FreeCAD [17]. To reconstruct the exact position of the cylinder relative to the rotation axis, the *Sketcher* workbench of FreeCAD (release 0.19) was used. Its general purpose is to create parameterized shapes in 2D that are used for the creation of 3D objects. This parameterized character is utilized to model the projection geometries of four orthogonal projections. With known geometrical values, such as distances, the diameter of the cylinder and its internal features, and the projection position on the detector screen, the exact position of the cylinder itself can be reproduced. This reproduction is crucial since any error of the virtual cylinder rotation and position relative to the center of the rotation table introduces new artifacts: the required shift of around 0.5 mm in X and Y directions (each) leads to a movement of the projected cylinder contours of 34 pixels (at a 0.4 mm pixel size) on the detector screen. The sinogram after a 40° MW simulation augmentation with incorrect positioning is shown in Figure 2.

The XCT Scan Module of aRTist can be used to automatically rotate the object after each projection and save all data in chronological order. If the underlying data includes a Missing Wedge problem, the real scan must be performed with a scan geometry that would allow for a sufficient rotation of the object if other objects or walls would not interfere. This needs to be considered when performing a real-world Missing Wedge scan with the intention of using simulation augmentation.

### 3.2. Combining Data—Augmented Sinograms

We acquired a full dataset containing no RoI or Missing Wedge limitations. This enabled the creation of a reference reconstruction and the mimicking of arbitrary RoI and Missing Wedge problems. To prepare a RoI dataset, one would specify a different width of the detector image (for 1D projections). During processing, the scan projection images were symmetrically cropped on both sides to obtain realistic RoI images. Widths of 75%, 50%, and 25% of the cylinder’s diameter were used. The cylinder’s projected width was around 870 pixels, and the truncated projections each covered 85.5%, 61.0%, and 31.4% of its cross-section area.

To prepare a Missing Wedge dataset, a certain angular range was cut from the full sinogram. This range was mirrored to the other side of the scan trajectory since a full scan over 360 degrees was available (note that typical Missing Wedges in electron tomography span over 40 degrees out of 180 degrees [18]). During the Missing Wedge simulation, it was calculated which projections lie inside the missing area. Such projections were chosen in a way that the orientation of the Missing Wedge was always vertical in the reconstruction image. The selected projections were ignored or replaced by black pixels (zero values) in the sinogram, depending on the use case.

RoI and MW cases could be simulated independently or in combination.

### 3.3. Computation Details

**Figure 3:**
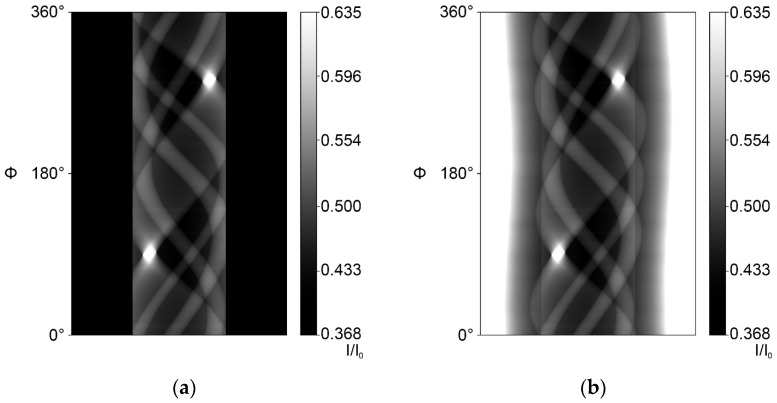
Intensity sinograms (transmission) of a 50% RoI case: (**a**) Incomplete 50% RoI scan intensity sinogram. (**b**) Simulation-augmented 50% RoI intensity sinogram.

**Figure 4:**
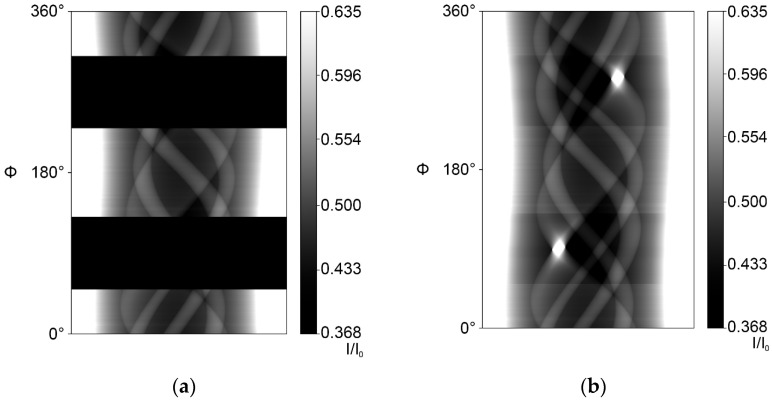
Intensity sinograms (transmission) of an 80° MW case: (**a**) Incomplete 80° MW scan intensity sinogram. (**b**) Simulation-augmented 80° MW intensity sinogram.

To combine the experimental data and the complete simulated projections, a custom image processor checked their compatibility and then used multiple image containers to compute (experimental) scan (Figure 3a and Figure 4a) sinograms, result sinograms (Figure 3b and Figure 4b) and projection files of both the incomplete dataset and the combined dataset.

During the data augmentation, all real and simulated projections were processed and combined into complete projections. These combined projections use as much real information as possible. First, result projections were created out of simulated data. Depending on the case of the current projection, the result data was replaced with real-scan data either completely, partially, or not at all. Three cases were defined: When no ROI or MW affects the real projection, its data completely replaces the simulated result data. If only a ROI is present in the real projection, the data inside the ROI partially replaces the simulated result data. If the real projection is not available (MW), no real data is taken, and the result projection stays completely simulated.

In the last step, the generated datasets were reconstructed with the CONRAD framework [11]. More detailed information on the computation can be found in the supplementary.

## 4. Results

**Figure 5:**
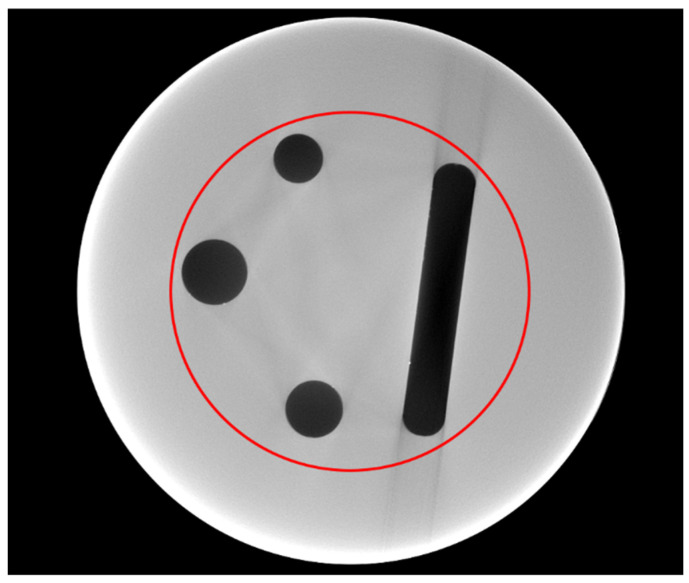
Reference reconstruction of the full dataset. The red circle indicates the area for the numerical evaluation (Section 5.2).

Figure 5 shows the reference reconstruction of the cylinder, generated from a complete dataset with 1500 projections over 360 degrees. A red circle marks the evaluation area used for the numerical evaluation. All reconstructions are shown in an identical grey scale. The 16-bit images were converted to 8-bit, setting a value scaling with 1300 as black (0) and 3700 as white (255). The only exceptions are the 50% RoI reconstruction, which has 1250 set as black and 28,000 as white, and the combined case (RoI & MW), with 0 as black and 40,000 as white.

**Figure 6:**
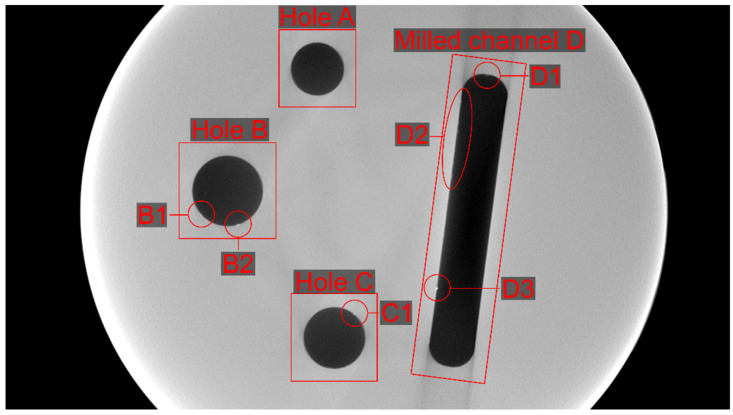
Indication of global features, i.e., the holes and the milled channel, and the labeled inclusions (B1 to D3) used for visual evaluation (see close-ups below).

Figure 6 labels all relevant global and local features in the reference FBP reconstruction of the experimental data. The holes were named counter-clockwise—A, B, C—and the milled channel D. Hole A features no inclusions, while Hole B has two inclusions, B1 and B2. Hole C has one inclusion, C1. The milled channel D has some material residue D1 at its upper end and one inclusion D3 in its lower third. In the upper third, the left side also features a series of residue particles named D2. 

### 4.1. Region-of-Interest Case

**Figure 7:**
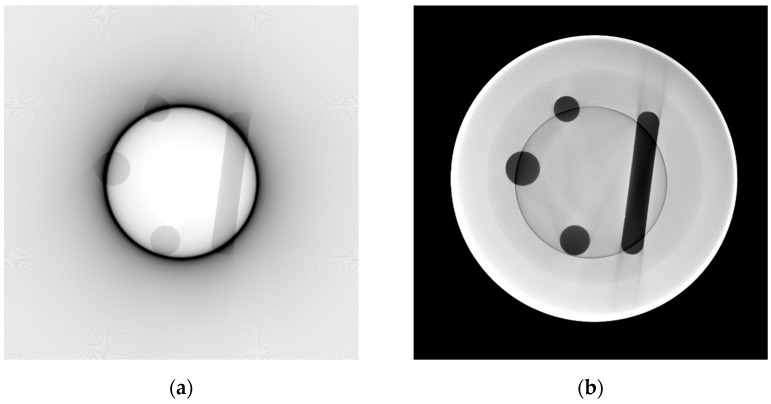
Reconstruction results were obtained from the 50% RoI data ((**a**), see sinogram data in Figure 3) and the augmented data (**b**).

For the Region-of-Interest case, a RoI size of 50% of the cylinder diameter was chosen. Its reconstruction can be seen in Figure 7. The area inside the RoI is covered by all projections, resulting in complete data. From the edge of the RoI towards the edge of the image, the number of projections that cover one voxel decreases, resulting in a typical lemon-shaped distortion towards the RoI.

At the edge of the RoI, a circle of low grey values can be observed. It creates a shine towards the inside and outside of the image, overlapping with the relevant information. This edge passes through holes A and B and the endings of the milled channel D. Due to the artifact ring shine; all inclusions show reduced contrast (see close-ups below). The inclusions B1 and D1 are located outside the RoI area and are difficult to recognize.

Simulation augmentation was able to improve inclusion visibility nearly to the quality of the reference reconstruction. Only the inclusions outside the RoI suffered from reduced contrast the further they were located from the RoI edge. Reconstruction results of the 75% and 25% RoI data can be found in the supplementary material of this paper (Figures S2–S4 therein).

### 4.2. Missing Wedge Case

**Figure 8:**
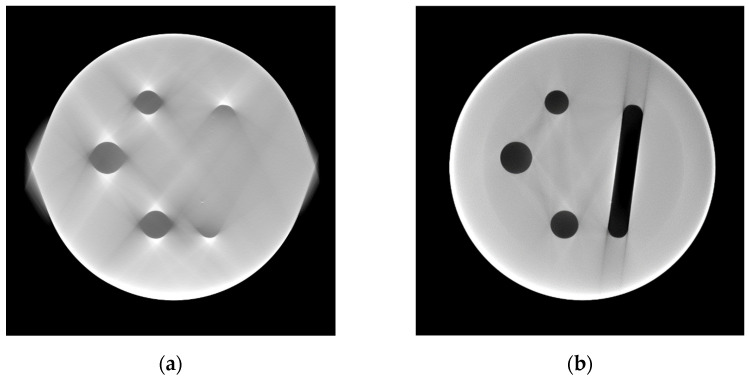
Reconstruction results were obtained from 80° Missing Wedge data ((**a**), see sinogram data in Figure 4) and the augmented data (**b**).

We chose a Missing Wedge of 80°. Figure 8 shows the reconstructions of the incomplete and simulation-augmented dataset. Obviously, the reconstruction of the incomplete dataset features warped geometries along the horizontal axis, which is perpendicular to the mean MW orientation. Edges oriented along the mean MW direction, such as the milled channel, completely disappear from the reconstruction. Even though the channel is barely visible, the inclusions D2 and D3 are easy to recognize, as they appear to be floating in the background (see close-ups below). 

After simulation augmentation, the warp artifact is mostly suppressed, but all inclusions possess reduced contrast with rising percentages of missing projections. The inclusion dampening occurs independent of the location. Still, the elimination of the warp artifact enables the allocation of previously floating inclusions at global features inside the scanned cylinder.

Reconstruction results of the 40° and 120° MW data can be found in the supplementary material section (Figures S5–S7 therein).

### 4.3. Combined Case: 50% Region-of-Interest + 40° Missing Wedge

**Figure 9:**
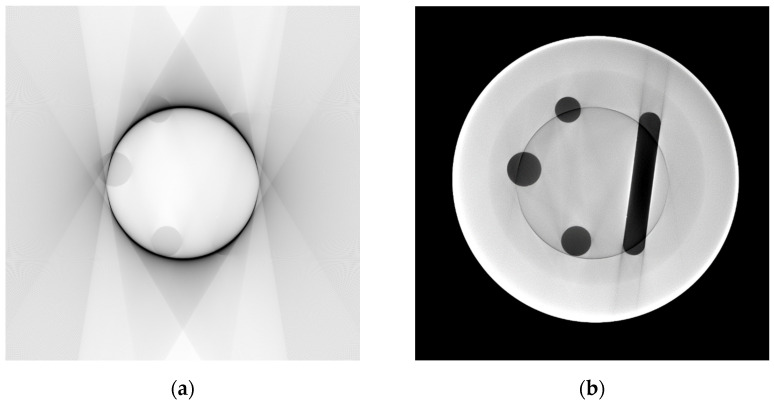
Reconstructions were obtained from 50% Region of Interest and 40° Missing Wedge experimental data (**a**) and augmented data (**b**).

**Figure 10:**
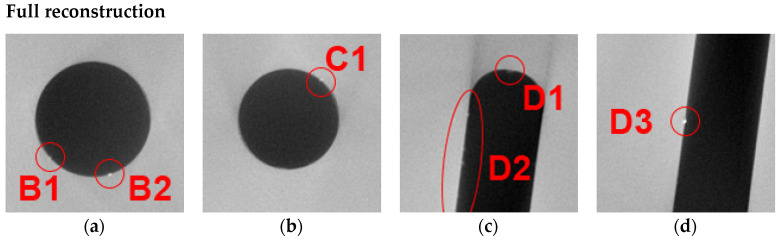

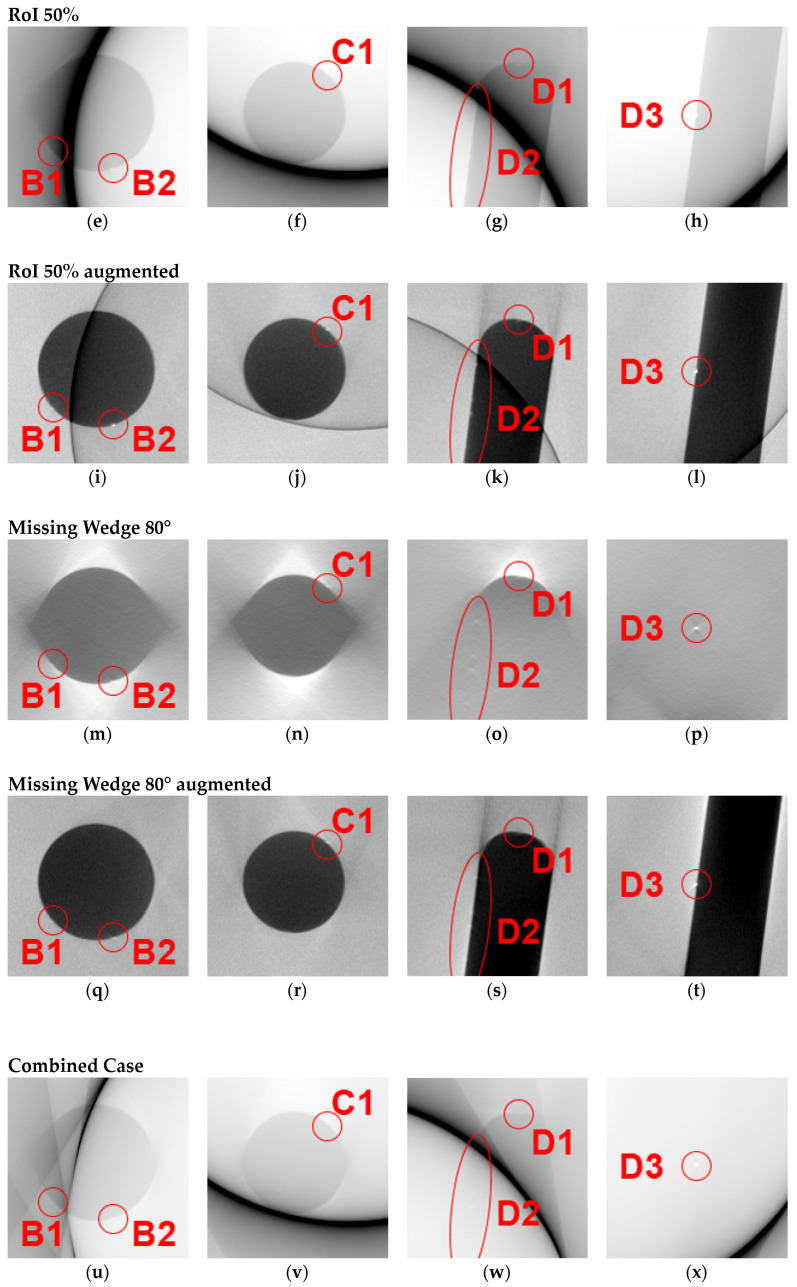

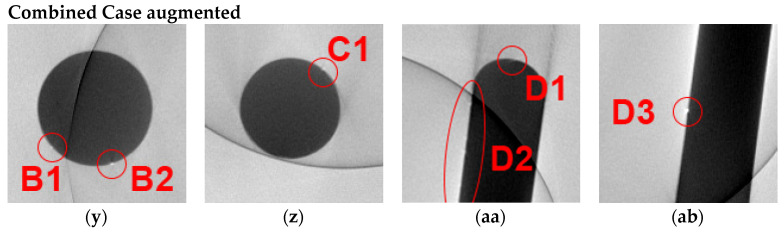
Close-up comparison of inclusion visibility of the full reconstruction (**a**–**d**, detail of Figure 6), of the (incomplete) 50% RoI dataset (**e**–**h**, detail of Figure 7a), of the augmented 50% RoI dataset (**i**–**l**, detail of Figure 7b), of the (incomplete) 80° MW dataset (**m**–**p**, detail of Figure 8a), of the augmented 80° MW dataset (**q**–**t**, detail of Figure 8b), of the (incomplete) combined 50% RoI + 40° MW dataset (**u**–**x**, detail of Figure 9a), of the augmented combined 50% RoI + 40° MW dataset (**y**–**ab**, detail of Figure 9b).

When combining RoI and MW, the problems seem to amplify their influence. The observed reconstruction of a 50% RoI and 40° MW (Figure 9) shows a degradation of the image quality. The milled channel inside the RoI completely disappears, and the visibility of all inclusions is greatly reduced (see close-ups in Figure 10u–x). After augmenting the dataset with simulated images (Figure 10y–aa), both the geometry and all inclusions are restored. In the incomplete dataset, the B1 and D1 inclusions were practically impossible to see and are well visible in the augmented reconstruction. The RoI artifact is still present but reduced, and there is almost no indication of MW artifacts.

## 5. Discussion

### 5.1. Visual Evaluation

Table 2 provides a summary of the qualitative assessment (visibility) of the geometries and their inclusions based on the following scale:

- 1: easy to recognize, almost as the reference
- 2: still easy to recognize, but geometry differs slightly from the reference
- 3: visible, but strong difference to reference
- 4: barely to see, and/or not recognizable structure
- 5: not useful at all

**Table 2:** Feature quality improvement of the visibility of global features and inclusions between incomplete and augmented RoI and MW reconstructions. The first grade refers to the incomplete data, while the number after the arrow is the grade of the augmented data reconstruction.

Feature	Reference	RoI 75%	RoI 50%	RoI 25%	MW 40°	MW 80°	MW 120°	Comb.
Hole A	1	2 → 1	2 → 1	4 → 1	2 → 1	3 → 1	3 → 1	4 → 1
Hole B	1	2 → 1	2 → 1	4 → 1	2 → 1	3 → 1	3 → 1	3 → 1
Hole C	1	2 → 1	2 → 1	4 → 1	2 → 1	3 → 1	3 → 1	3 → 1
Channel D	1	2 → 1	3 → 1	4 → 2	4 → 1	5 → 1	5 → 1	5 → 1
B1	1	3 → 1	4 → 1	5 → 5	1 → 1	3 → 3	4 → 5	4 → 2
B2	1	2 → 1	2 → 1	4 → 3	1 → 1	3 → 3	3 → 4	3 → 1
C1	1	3 → 1	2 → 1	4 → 2	1 → 1	3 → 3	4 → 4	3 → 2
D1	1	4 → 1	4 → 2	5 → 5	1 → 2	3 → 3	4 → 4	5 → 1
D2	1	2 → 1	2 → 1	4 → 3	1 → 2	1 → 3	1 → 4	3 → 2
D3	1	2 → 1	2 → 1	3 → 2	1 → 1	1 → 1	1 → 2	3 → 1

In all cases, the overall geometry of the object is improved up to perfect visibility (value = 1). This is expected because the simulated data mimics the real object. For the RoI cases, the simulation augmentation always improved the visibility of the features, though to a lesser extent in extreme situations, for instance, if an inclusion was previously not visible at all. Interestingly, in the Missing Wedge cases, the simulation augmentation sometimes led to a degradation of the visibility. This phenomenon can be best showcased by the inclusion of D3 in the MW 80° case (Figure 10p,t) (even though the visibility grading shows no improvement or degradation of visibility). In the MW 80° reconstruction, the geometry of the milled channel D is almost completely lost, while the inclusion appears to be floating inside the solid material. The reason for this is that the approximately circular inclusion always features contours that align with the direction of the Missing Wedge, while the channel does not. Since the inclusion reconstruction still has a high contrast to the background, it can easily be seen in the slice reconstruction, even though its location inside the object is much harder to identify. In the augmented reconstruction, though, the channel geometry is restored back to reference data quality, and the inclusion position is unambiguous. Unfortunately, in such a case, a degradation of visibility occurs since the simulated XCT dataset does not feature this inclusion and partially overwrites it, leading to a lower contrast in the reconstruction.

### 5.2. Numerical Evaluation

To support the subjective evaluation results with quantitative data, several numerical evaluation metrics were applied. Here, each pixel or window of pixels of an incomplete and an augmented reconstruction is compared to the corresponding pixel or window of the full reconstruction, which, therefore, acts as a reference.

The first and simplest metric is the Root Mean Square Error (RMSE) [13]

(1) $$RMSE_{X,Y}\;=\;\sqrt{\sum_{i\;=\;1}^{n}\frac{{\left(x_{i}\;-\;y_{i}\right)}^{2}}{n}\;}.$$

Alternatively, the Pearson Correlation Coefficient (PCC) [14,15] was used.

(2) $$PCC_{X,Y}\;=\;\frac{\sum_{i=1}^{n}\left(x_{i}-\bar{x}\right)\cdot \left(y_{i}-\bar{y}\right)}{\sqrt{\sum_{i=1}^{n}{\left(x_{i}-\bar{x}\right)}^{2}}\cdot \sqrt{\sum_{i=1}^{n}{\left(y_{i}-\bar{y}\right)}^{2}}}.$$

Each pixel is compared with the corresponding reference pixel. In Equations (1) and (2), *X* and *Y* are two images, $x_{i}$ and $y_{i}$ are single pixels from *X* and *Y* and $\bar{x}$ and $\bar{y}$ the mean values of all pixels out of *X* and *Y*, respectively. *n* pixels are present in each image.

The PCC gives information about the correlation of pixel values, $x_{i}$ and $y_{i}$, between two images. Here, a grey value mismatch between pixels has no effect on the result if such a mismatch is constant. A value of PCC between 0.70 and 1.0 shows a very strong positive correlation, while a value between −0.70 and −1 would show a very strong anti-correlation. A value of zero shows that no correlation of the images is present.

Additionally, the Structural Similarity Index Metric (SSIM) [16] between reconstruction results and the reference was calculated. The SSIM improves upon the PCC by comparing the structure “after luminance (grey value) subtraction and variance normalisation” [16] and using the results of independent grey value and contrast (variance) comparisons. Since grey value and contrast may change across the image and should have a minimal effect on the result, this metric works in small windows instead of the whole image at once. Due to the complexity of the SSIM metric, its further definition details are not mentioned here and can be found in the work by Wang et al. [16].

When comparing a reconstruction and the reference, PCC and SSIM values close to 1 indicate that, overall, the reference strongly resembles the original image. It should be noted that these metrics are mostly applicable to the quality of the overall geometry since they compare the structure of the whole image, not just local feature visibility. Inclusions have a small influence on the results since they are small compared to the other structures in the image.

The comparison of the reconstruction of a fully simulated XCT scan of the acrylic cylinder to the experimental scan results in a PCC value of 0.9882 and an SSIM of 0.9887. They all indicate a very strong correlation. A perfect 1.0 cannot be achieved because of

- the different geometry of the virtual cylinder, compared to the experimental one. This factor has a rather strong influence because, at the edges of the global features, the simulation could show 1–2 voxels of material where there is actually no material left (or vice-versa). Such outliers have a strong influence on the PCC and SSIM’s structure comparison.
- the absence of any inclusions inside the simulation. This absence leads to further outliers. Since it only produces a few of them, its influence on the PCC and SSIM is rather weak.
- the energy sensitivity curve of the virtual detector. Such a sensitivity curve needs to be adjusted by hand to match the measured grey values. This adjustment is never perfect and results in a grey value shift at certain intensities (see, e.g., Figure 8, right). Because the grey values of the simulated reconstructions are consistent with the experimental data, the impact on the PCC is rather small and should be even smaller on the SSIM since the grey value intensity is evaluated independently.

For the numerical evaluation, only a relevant area of internal geometry was taken into account. It was chosen as small as necessary to minimize the overall pixel count (see Equations (1) and (2)). However, such a choice causes the grey value mismatch at the inclusions and artifacts around the removed material to have a stronger influence on the result. The area covers all pixels inside a circle, with its center located at the cylinder sample center. The radius is chosen such that the holes and the milled channel are fully inside, and a slight margin at the edge is left. In Figure 5, the red circle marks the edge of the observed area. Pixels inside the area are compared to the reference, while pixels outside it are disregarded. The numerical evaluation results for the three different metrics are listed in Table 3.

The RMSE results mostly behave intuitively, where smaller RoI and larger MW lead to higher error values. All RoI cases have significantly higher values than MW because the reconstructed images feature the bright ring artifact, and the area outside the RoI greatly differs from the reference. Moreover, all figures-of-merit decrease with increasing complexity (i.e., with an increasingly large fraction of missing data). One exception from the intuitive behavior is the RoI 50% case, which has much larger values of all metrics than the RoI 75% and RoI 25%. This behavior can be explained with geometrical considerations. In the 75% RoI, the artifact circle lies outside the analyzed area and has no direct impact on the result. In the RoI 50% case, this circle is right inside the observed area and occupies more pixels than the artifact circle of the RoI 25% case.

Both the PCC analysis and the SSIM analysis show the same behavior.

Augmenting the experimental dataset with simulated projection data always improves the result, bringing the image quality nearly to that of the reference image, in spite of the correlation between the extent of missing experimental data and the result value of the augmented reconstruction.

## 6. Summary

The proposed approach shows that simulated XCT data can be used to greatly improve the FBP reconstruction quality of incomplete Region of Interest and Missing Wedge XCT datasets. While the simulation augmentation fully restores the overall geometry of an object, small deviations to the simulation (typically material or construction defects) suffer from a dampening in contrast relative to their background. The effectiveness of the approach decreases with an increasing percentage of missing information in the experimental scan.

Inside a RoI, where the complete information is available, the data augmentation mostly results in a strong reduction of the common ring artifact and its shine inside the RoI. This leads to greatly improved image quality towards the inner edge of the RoI area. For the RoI problem, simulation augmentation improved the visibility of inclusions in all observed cases. The overall internal geometry of the object was always restored, and all features could be retrieved. Different RoI sizes down to 25% of the object diameter were tested. This case shows that inclusions that are not visible in an incomplete reconstruction also do not appear in the reconstruction of augmented data. Also, the typical RoI ring artifact could not be completely removed due to an imperfect simulation. Its impact on the whole image (a circle of low grey values) was greatly reduced, and its core brightness was reduced from 65,000 to 3000 grey values (in the 25% RoI case).

In the Missing Wedge case, the overall image geometry could be greatly improved. In unfavorable cases of MW, where large parts of the global features completely disappeared in the classic reconstruction, such features could be recovered. In the classic reconstruction, inclusions that were located at the disappeared contours of channel D were easier to see than in a reference reconstruction (full 360° dataset). Since these inclusions could not be allocated to a specific geometry, the benefit of this effect is questionable. The contrast of these inclusions was slightly decreased in an augmented reconstruction, but their location in the object could be precisely identified. The warping artifacts caused by MW could almost completely be removed by replacing the missing projections with simulated ones.

## 7. Conclusions

The quality enhancement of augmented FBP reconstructions offers the possibility of using RoI and Missing Wedge datasets with almost no artifact influence. However, the required manual labor when creating a simulated XCT scan still reduces the applicability to real-world cases. Even with an accurate 3D model of the scanned object, the model needs to be perfectly positioned inside a virtual XCT scanner. Additionally, this scanner needs to be set up in a way that it closely mimics the real-world device. The initial setup is based on a priori information, such as the XCT scanner metadata and the documentation of the scan; however, such information needs to be updated at a later stage based on the results of preliminary reconstructions of the created dataset. Both the initial setup and the fine adjustments have the potential to be automated, leading to improved real-world applicability. If only a prototype of the object, i.e., general structural characteristics, is known, the approach would have to be transformed into an iterative algorithm for precise positioning and refinement of the individual (local) structure.

With reduced manual labor, the approach of simulation-augmentation has the potential to improve other reconstruction algorithms than FBP. It also opens up the way to compare two simultaneously reconstructed images: the original reconstruction of incomplete data and the other one, a simulation-augmented image. In the original image, some inclusions may be significantly easier to see (e.g., in some MW cases), while the augmented reconstruction offers the possibility to evaluate the inclusion in the context of a correct object geometry.

## Figures and Tables

**Table 1:** Technical details of the XCT system used.

Scanner	GE phoenix v|tome|x L300
X-ray tube	xs|180 HPNF (high power nanofocus) operated at 60 kV and 150 mA, tungsten target, no filter
Detector	GE dynamic 41|200 with 2048 × 2048 pixel resolution, 200 μm pixel size, and 2 × 2 binning
Source to detector distance	450 mm
Source to object distance	55 mm
Magnification	8.18
Effective voxel size	48.9 µm

**Table 3:** Results of RMSE, PCC, and SSIM comparisons between incomplete/augmented reconstructions and the reference.

	RMSE		PCC		SSIM	
	Incomplete	Augmented	Incomplete	Augmented	Incomplete	Augmented
ROI 75%	1123	148	0.8540	1.000	0.7482	0.9966
ROI 50%	11,564	150	0.1794	0.9847	0.5335	0.9954
ROI 25%	7704	177	0.0389	0.9783	0.7323	0.9949
MW 40°	407	73	0.7326	0.9937	0.9890	0.9995
MW 80°	519	83	0.5448	0.9916	0.9829	0.9964
MW 120°	703	96	0.4166	0.9902	0.9664	0.9945
RoI 50% & MW 40°	9843	168	0.1082	0.9848	0.6117	0.9952
Simulation	280		0.9882		0.9887	
Optimum	-		1		1	

## Data Availability

Any data used in this paper is available from the authors on reasonable request.

## Supplementary Materials

The following supporting information can be downloaded at: https://www.mdpi.com/article/10.3390/jimaging10010011/s1, Figure S1: detector characteristics curve; Section S2: computational details of merging real and simulated data; Figure S2: reconstructions of 75% RoI incomplete and augmented dataset; Figure S3: reconstructions of 25% RoI incomplete and augmented dataset; Figure S4: close-up comparison of insert visibility for the 75% and 25% RoI cases; Figure S5: reconstructions of 40° MW incomplete and augmented dataset; Figure S6: reconstructions of 120° MW incomplete and augmented dataset; Figure S7: close-up comparison of insert visibility for the 40° and 120° MW cases.
