# Supplementary material for: Simulation-Assisted Augmentation of Missing Wedge and Region-of-Interest Computed Tomography Data
Source: J Imaging. 2023 Dec 29;10(1):11. doi: 10.3390/jimaging10010011 (PMC10817004; doi:10.3390/jimaging10010011)
Supplement: Supplementary file 1 [file jimaging-10-00011-s001.zip › jimaging-2663842-supplementary.pdf]

Supplementary Material

Simulation-Assisted Augmentation of Missing Wedge and Region-of-Interest Computed Tomography Data

by Vladimir O. Alekseychuk, Andreas Kupsch, David Plotzki, Carsten Bellon
and Giovanni Bruno

This supplementary material contains supporting background information, which does not fit into the original paper. We show the non-linear detector characteristics curve (section 3.1), we give detailed information on the computation of merging real and simulated data (section 3.3), and we show reconstruction results of different degrees of Region of Interest and Missing Wedge data, which are referred to in the evaluation sections 5.1 and 5.2, but not shown in the respective reconstruction results sections 4.1 and 4.2.

S1 Detector characteristics curve

(supporting section 3.1)

Figure S1 shows the used characteristic curve of the simulated detector. It describes which energy intensities (in units of J/m²) are converted to which grey value in each pixel.

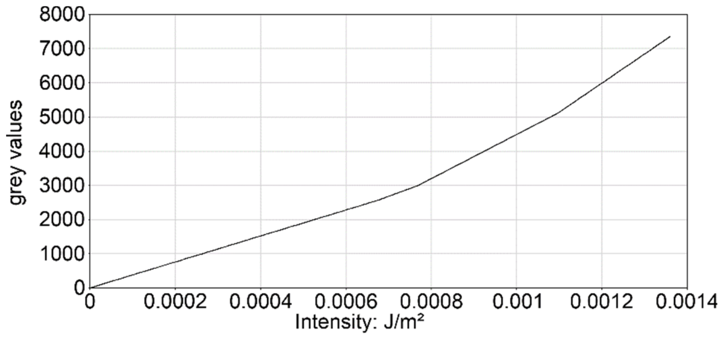

Figure S1. Characteristic grey value curve of the simulated detector. X-ray intensities (J/m²) are converted to grey values.

S2 Computation details – Merging real and simulated data

(supporting section 3.3)

To actually combine the real data and the complete simulated projections, the processing program first needs to check their compatibility in terms of:

- **Projection size:** All projections should be in 1D and the real experimental projections should never be wider than the simulation, as this defeats contradicts the purpose of simulating data in a RoI context. A function should be available to convert 2D projections into 1D by only using a specified line of the image. When saving the 1D data, it should also be ensured that file counting starts at 0.

- **File naming:** The naming of the projection files should allow for automatised iteration. The processing program was designed to follow a syntax of NAME_NUMBER and start counting at 0.
- **Projection count:** The number of projections for the real and simulated data should be equal. It is assumed that when creating the simulation data, it covers the same angle as the real data (in this case 360 degrees).

Then, several image-containers are created that hold the following data:

- **Scan projection container:** The currently used scan projection with number *i*
- **Simulated projection container:** The currently used simulated projection with number *i* (same as scan projection number)
- **Result projection container:** The augmented projection that has the number *i*, as well
- **Scan sinogram:** The sinogram of the scan data after applying RoI and/or Missing Wedge scenarios (Figure 3a, Figure 4a in the main text).
- **Simulation sinogram:** The sinogram of the full simulated data.
- **Result sinogram:** The sinogram includes the combined data of the incomplete real scan and the complementary simulated scan (Figure 3b, Figure 4b in the main text).

The program iterates through all projections and during each iteration, the corresponding real and simulated projections are loaded into their respective containers. After that, the result projection is filled with data from the simulation projection. The simulated data is later overwritten with the real scan data, depending on the present case:

- **No restrictions:** All pixels of the scan data are copied into the target projection and replace all previously copied data from the simulated projection. This case occurs, when no RoI is present and either no Missing Wedge is applied or if the projection is not affected by MW.
- **Region of interest:** For the RoI case, the previously set RoI width x is used. x pixels from the center of the projection are taken and copied to their respective position in the result projection (overwriting a part of the simulation data). This leads to a projection that has simulated data at the detector boundaries and real data in its center. The x center pixels of the real projection are also copied to the line in the real scan sinogram that represents this projection. The width of the real scan sinogram equals x.
- **Missing Wedge:** If the real projection is missing, the result projection is not modified and still consists completely of simulated data. A line of black pixels is added to the real scan sinogram, where the missing projection should be.

The resulting projection is also copied to its correct line in the result sinogram.

Each resulting projection and RoI and MW-processed real scan projection is saved, to have input data for an incomplete and augmented reconstruction. After all iterations, the sinogram images are saved as well, to enable a first evaluation of the results, without having to perform a reconstruction. Figures 3 and 4 (in the main text) display the sinogram outputs before and after augmenting 25% RoI data and 80° Missing Wedge data.

S3 Reconstruction results

S3.1 Region of Interest

(supporting section 4.1)

Figures S2 and S3 show reconstructions of Region of Interest cases with 75% RoI and 25% RoI that were not covered in the main article. The left image shows the reconstruction of incomplete data and the right image shows the reconstruction of the simulation-augmented data. For the conversion from 16-bit to 8-bit grey scale, 1300 was set as black (0) and 3700 as white (255) in the simulation-augmented reconstructions. The incomplete 75% RoI reconstruction (Figure S2 left) uses 600 as black and 15000 as white. The incomplete 25% RoI reconstruction (Figure S3 left) uses 0 as black and 22300 as white. Figure S4 illustrates the reconstruction of the small details B1 to D3 under the two RoI restrictions in analogy to Figure 10 in the main text.

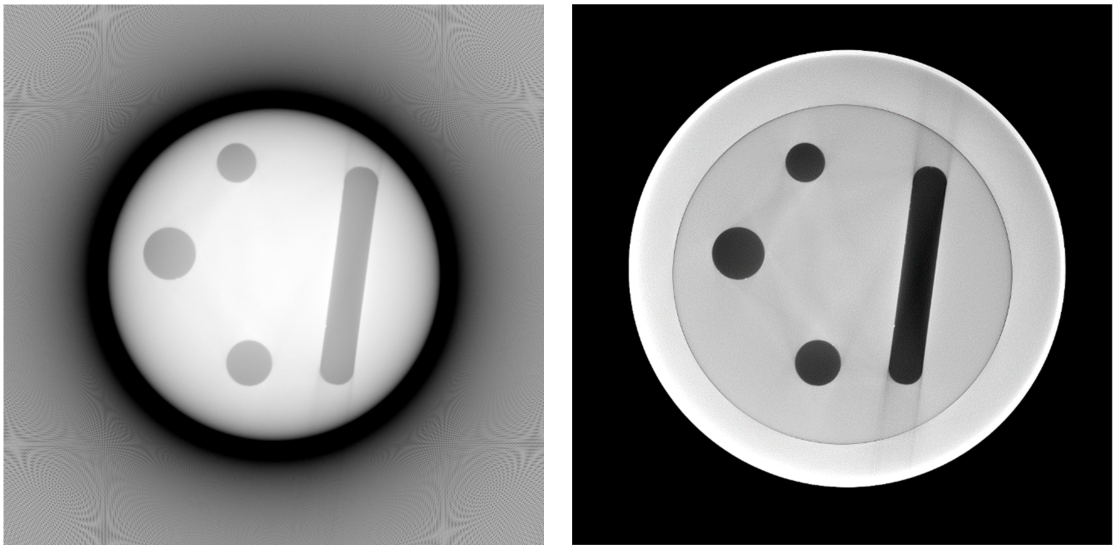

Figure S2. Left: reconstruction of an incomplete 75% RoI dataset, right: reconstruction of an augmented 75% RoI dataset.

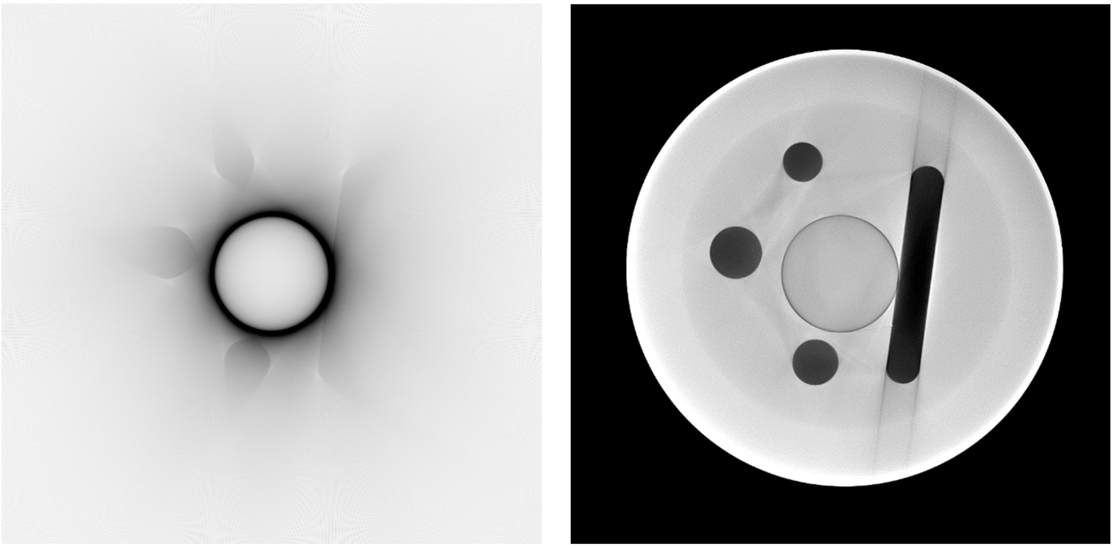

Figure S3. Left: reconstruction of an incomplete 25% RoI dataset, right: reconstruction of an augmented 25% RoI dataset.

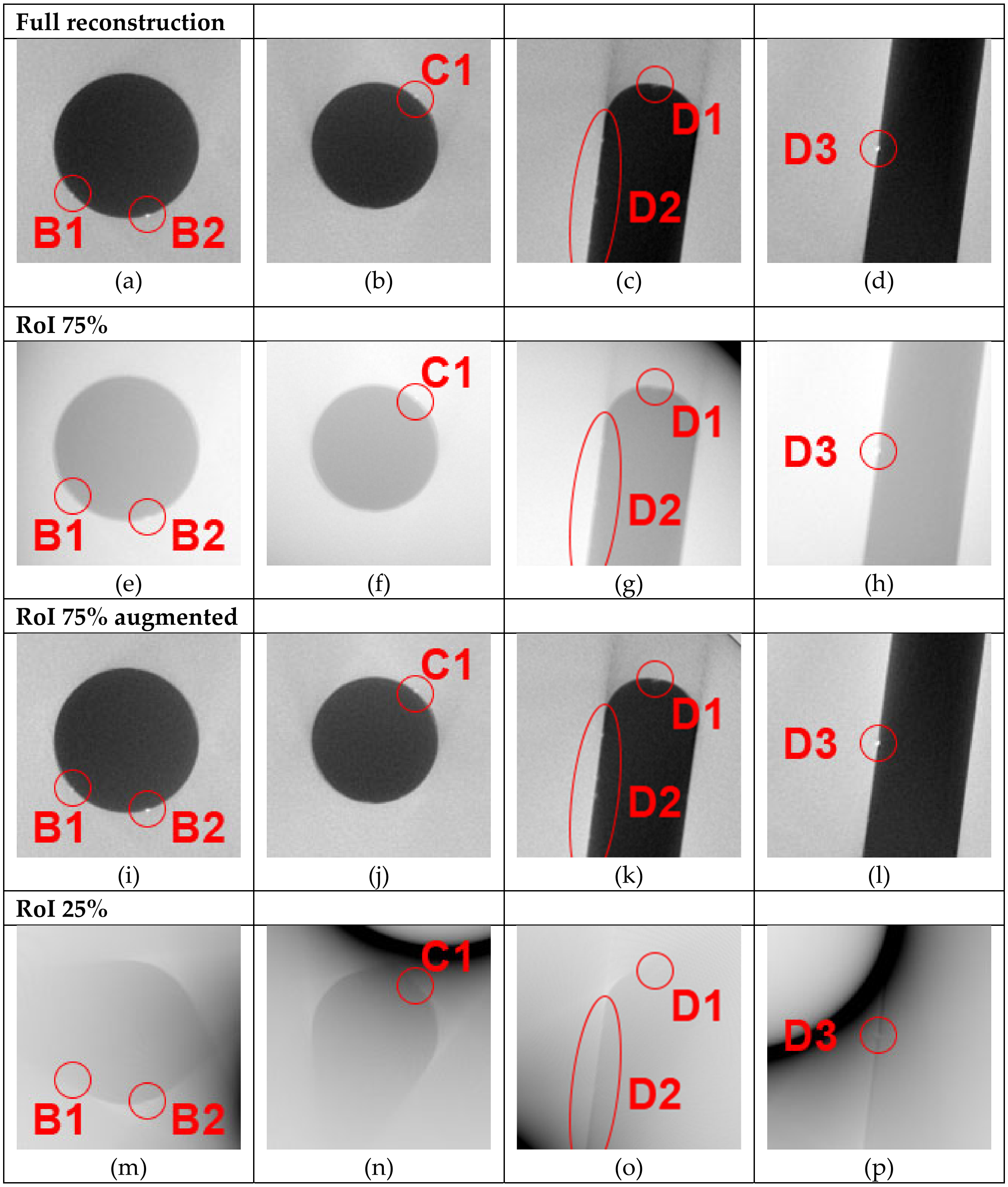
Full reconstruction
B1
B2
(a)
C1
(b)
D1
D2
(c)
D3
(d)
RoI 75%
B1
B2
(e)
C1
(f)
D1
D2
(g)
D3
(h)
RoI 75% augmented
B1
B2
(i)
C1
(j)
D1
D2
(k)
D3
(l)
RoI 25%
B1
B2
(m)
C1
(n)
D1
D2
(o)
D3
(p)

RoI 25% augmented

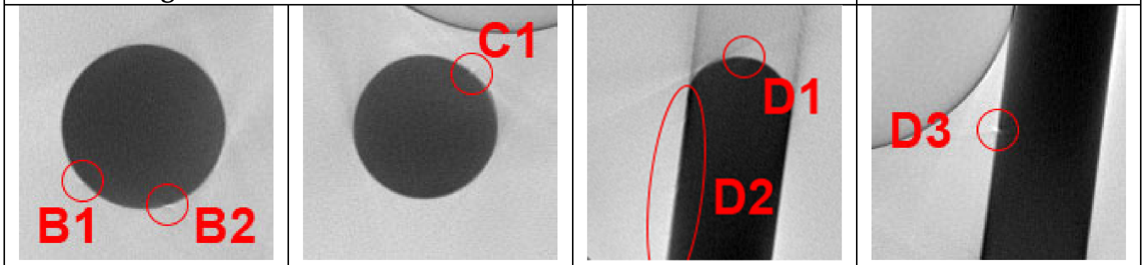

(q) (r) (s) (t)

Figure S4. Close-up comparison of insert visibility for the 75% and 25% RoI cases.

S3.2 Missing Wedge

(supporting section 4.2)

Figures S5 and S6 show reconstructions of Missing Wedge cases with 40° MW and 120° MW that were not covered in the main article. The left image shows the reconstruction of incomplete data and the right image shows the reconstruction of the simulation-augmented data. For the conversion from 16-bit to 8-bit grey scale, 1300 was set as black (0) and 3700 as white (255) for all reconstructions. Figure S7 illustrates the reconstruction of the small details B1 to D3 under the two MW restrictions in analogy to Figure 10 in the main text.

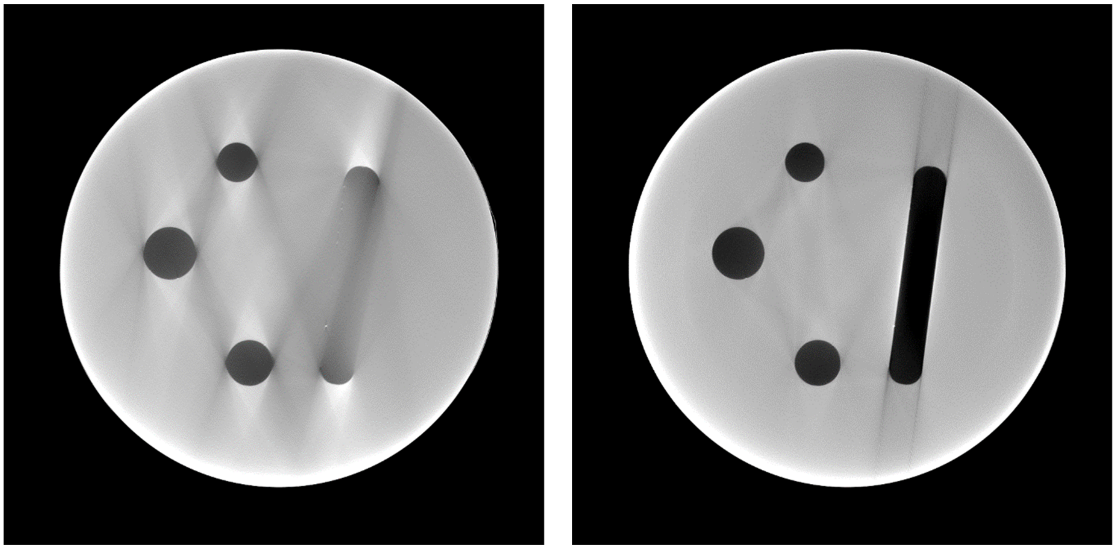

Figure S5. Left: reconstruction of an incomplete 40° Missing Wedge dataset, right: reconstruction of an augmented 40° Missing Wedge dataset.

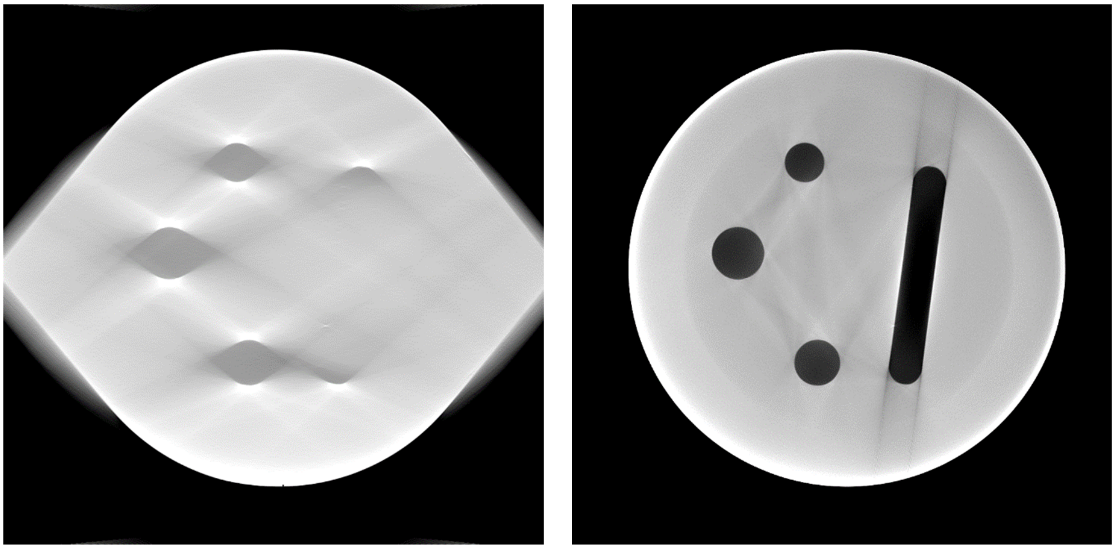

Figure S6. Left: reconstruction of an incomplete 120° Missing Wedge dataset, right: reconstruction of an augmented 120° Missing Wedge dataset.

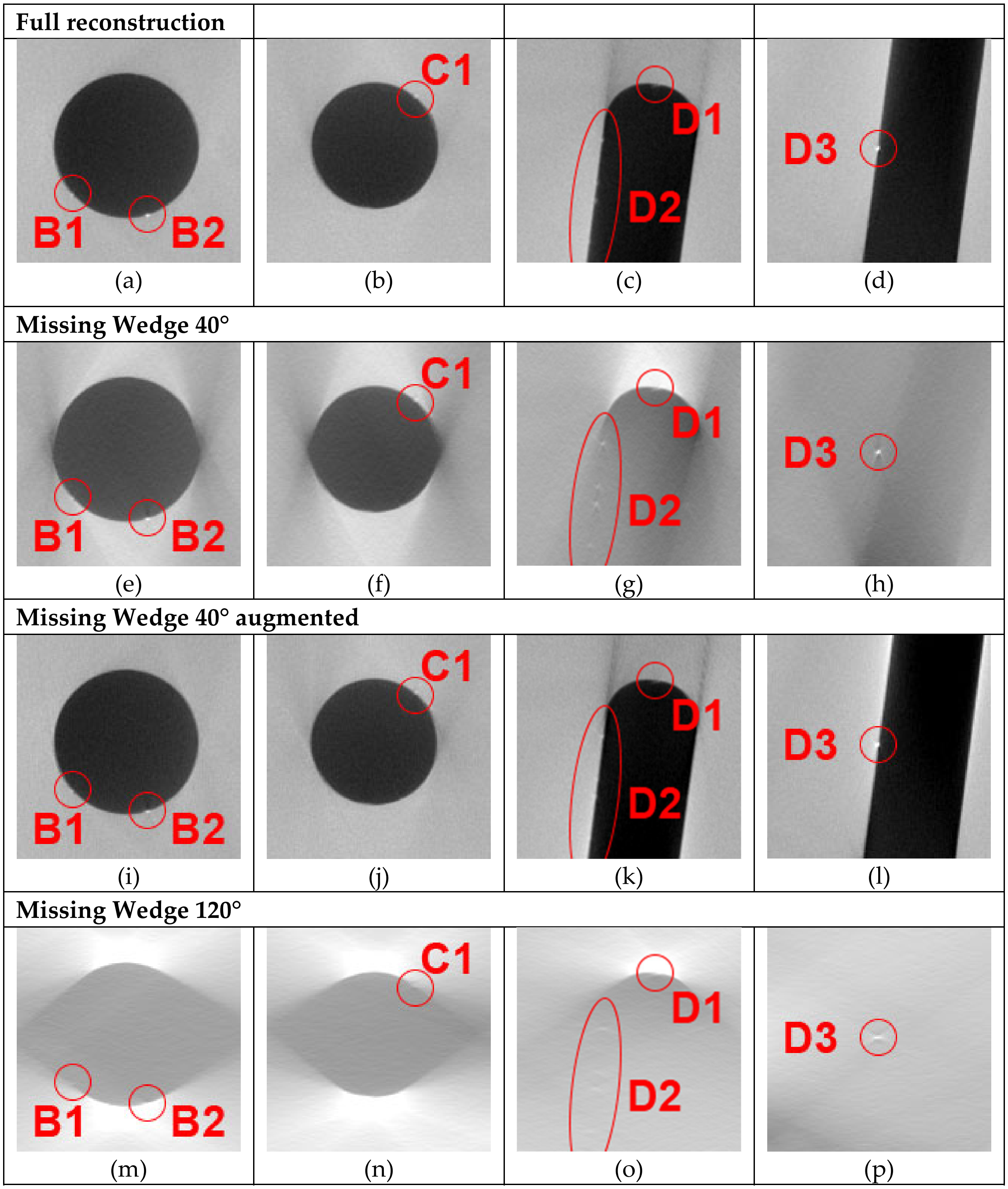

Full reconstruction
B1
B2
(a)
C1
(b)
D1
D2
(c)
D3
(d)
Missing Wedge 40°
B1
B2
(e)
C1
(f)
D1
D2
(g)
D3
(h)
Missing Wedge 40° augmented
B1
B2
(i)
C1
(j)
D1
D2
(k)
D3
(l)
Missing Wedge 120°
B1
B2
(m)
C1
(n)
D1
D2
(o)
D3
(p)

Missing Wedge 120° augmented

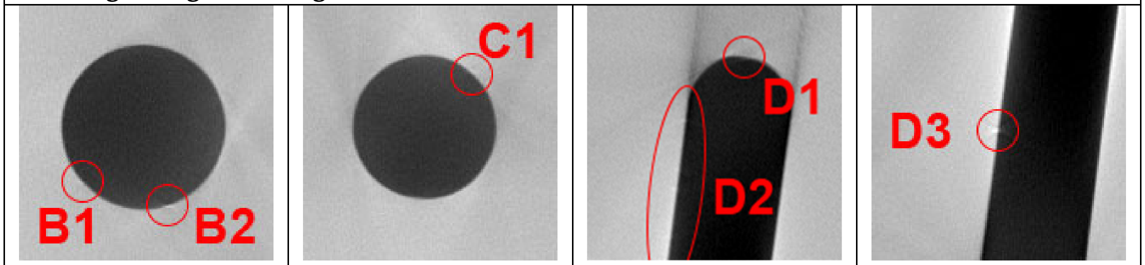

(q) (r) (s) (t)

Figure S7. Close-up comparison of insert visibility for the 40° and 120° Missing Wedge cases.
